# What Can Surrogate Tissues Tell Us about the Oxidative Stress Status of the Prostate? A Hypothesis-Generating In-Vivo Study

**DOI:** 10.1371/journal.pone.0015880

**Published:** 2010-12-28

**Authors:** Kaitlyn F. Whelan, Jian-Ping Lu, Eduard Fridman, Alex Wolf, Alon Honig, Gregory Paulin, Laurence Klotz, Jehonathan H. Pinthus

**Affiliations:** 1 Department of Surgery, McMaster University, Hamilton, Ontario, Canada; 2 Department of Surgery, University of Toronto, Toronto, Ontario, Canada; Florida International University, United States of America

## Abstract

**Background:**

Prostatic oxidative stress (OS) is androgen-regulated and a key event in the development of prostate cancer (PC). Thus, reducing prostatic OS is an attractive target for PC prevention strategies. We sought to determine if the individual's prostatic OS status can be determined by examining the OS in surrogate androgen regulated tissues from the same host.

**Methodology/Principal Findings:**

Adult male rats were divided equally into three groups: (A−) underwent bilateral orchiectomy, (A+) received continuous testosterone supplementation or (C) were eugonadal. Serum testosterone, 8-hydroxy-2-deoxyguanosine (8-OHdG) and anti-oxidative capacity (AOC) were determined after 72 hrs and the prostate, salivary glands and the hair follicles' Dermal Papillary Cells (DPC) from each animal were harvested, embedded into tissue microarray and examined for the expression of 8-OHdG by immuno-staining. Multi-variate regression was used to analyze inter-individual differences in OS staining within each androgen group and if there was a correlation between serum testosterone, 8-OHdG or AOC and Prostatic OS in tissues of same host.

At the group level, 8-OHdG staining intensity directly correlated with serum testosterone levels in all three target tissues (p>0.01, Mann-Whitney Test). Although different levels of prostatic OS were noted between rats with similar serum testosterone levels and similar systemic OS measurements (p<0.01), there were no intra-individual differences between the OS status of the prostate and DPC (p<0.05).

**Conclusions/Significance:**

The level of prostatic OS is correlated with the OS of hair follicles and salivary glands, but not systemic OS. Moreover, systemic AOC negatively correlates with both prostatic and hair follicle OS. This suggests that hair follicle and salivary gland OS can serve as surrogate markers for the efficiency of OS reduction. This has tremendous potential for the rational evaluation of patient response to prevention strategies.

## Introduction

Oxidative stress (OS) is a key event in the development of prostate cancer (PC) [1], [2], [3], its progression [3], [4], [5], [6] and its response to therapy [7]. OS is the result of an imbalance between the production of reactive oxygen species (ROS) and their detoxification. ROS are inevitable byproducts generated by cell metabolism and homeostasis, and have been shown to exert regulatory roles in controlling cell proliferation, survival, and differentiation [8] However, the accumulation of ROS, a typical phenomenon of PC [5], triggers a host of pro-carcinogenic processes. More specifically, increased levels of ROS can cause oxidative DNA damage. Furthermore, elevated levels of ROS cause enhanced cell signaling and activation of target genes in PC cells that promote cellular survival and progression [4], [9].

Inflammation induced OS has been associated with prostate carcinogenesis [5]. Local generation of ROS can be initiated by an environmental exposure to infectious agents, [1] dietary carcinogens, and hormonal imbalances that promote intra-prostatic inflammatory reactions [10]. Intra-prostatic OS, a phenomenon regulated via androgen receptor mediated pathways [7], is a prime target of PC prevention strategies [2], [11].

The physiological effects of androgens derive not only from their serum/tissue levels, but also from interaction with their receptors and unique activity per individual. The specific effects of androgens in tissues depend not only on the levels of androgens and expression of the androgen receptors, but also on individual variations in the activity of the androgen/androgen-receptor complex. For example, androgen-regulated activities are attenuated corresponding to the length of triplet CAG residues in the androgen receptor gene which is subjected to polymorphism [12]. The effect of androgens on OS in target tissues can not be predicted in an individual simply by measuring circulating androgen levels.

PC is an attractive candidate for prevention strategies because it has a high incidence, a protracted latent period and defined risk factors. Despite recent enthusiasm for micronutrient supplementation in the treatment of PC, recent research has called the efficacy of such practices into question. Both the Selenium and Vitamin E Cancer Prevention Trial (SELECT) and the Physician's Health Study II showed negative results for vitamin E, vitamin C, and selenium supplementation on decreasing PC risk [13], [14]. Since no individual baseline OS measurements were taken, the negative results from these studies could reflect the failure of a mass approach to prevention. Surrogate markers that can predict an individual's prostatic OS would complement a more rationalized selection of candidates for PC prevention.

The hair follicles and salivary glands are exocrine glands that express the androgen receptor and share common morphological characteristics with the prostate [15]–[18]. We thus hypothesize that the magnitude of basal OS in these tissues will correlate with that of the prostate in each individual. In this pre-clinical hypothesis-generating *in-vivo* experimental study of male rats, we sought to demonstrate that the status of prostatic OS correlates with levels in the hair follicles and the salivary glands of the same subject over and beyond its androgenic milieu.

## Materials and Methods

### ***In-Vivo*** Studies

After obtaining approval from the Animal Research Ethics Board at McMaster University (Permit # 08-09-40), eighteen adult Sprague Dawley male rats (SPF) were purchased from Charles River Laboratories. Each rat weighed between 200 and 250 grams and was randomized into one of three groups (each group contained 6 rats). The first group underwent a bilateral trans-scrotal orchiectomy to induce androgen deficiency and was labeled “A−” to indicate their lack of endogenous and exogenous androgens. Each rat in the second group underwent sham trans-scrotal procedure and sub-cutaneous implantation of a slow release testosterone pellet (12.5 mg/pellet; Innovative Research of America, Sarasota, FL). This group was assigned the label “A+” to indicate implantation the presence of excess androgens (both endogenous and exogenous androgens). The third group labeled “C,” to indicate this was the control group, underwent only a sham trans-scrotal operation and thus had no manipulation of the natural endogenous production of testosterone. All operative procedures described above were performed under ketamine + xylazine (127.5 and 4.5 mg/kg, respectively) general anesthesia. All of the animal handling, anesthesia, surgery, recovery and euthanasia were performed following the Standard Operating Procedures of McMaster University Central Animal Facility.

### Tissue Preparation

The rats were sacrificed 72 hours after hormone treatment. Blood was immediately withdrawn from the left ventricle and separated to serum by centrifugation and freezein-20°C until analysis. Tissue from the skin, prostate and salivary gland was immediately removed and fixed in 4% buffered formaldehyde.

### Immunohistochemistry

The fixed tissues were embedded in paraffin and then re-constructed into a pre-planed tissue micro-array (TMA) platform. Tissue array was made with hand-tissue-arrayer (Beecher Instruments, Sun Prairie, WI) with the core size set at 2 mm. The tissue arrays were then pretreated with proteinase K (DakoCytomation, Glostrup, Denmark) and washed with phosphate-buffered saline tween-20 (PBST). Normal Rabbit Serum diluted in 2% bovine serum albumin (BSA) was used to block the TMA's which were subsequently incubated for 30 minutes. The TMA's were then incubated with goat anti-8-hydroxy-2′-deoxyguanosine (8-OHdG) antibodies (1∶200; Chemicon International) for one hour. Biotinylated rabbit anti-goat IgG (Vector Laboratories, Burlingame, CA) was added for detection purposes and the TMA's were incubated for an additional 30 minutes.

### Tissue Assay

TMA sections were sequentially put though xylene, 100% ethanol, 95% ethanol, 50% ethanol and distilled water to deparaffinize and rehydrate them. Antigen unmasking of the sections were carried out according to the protocols of the antibody makers. Immunoreactions of the section were undertaken in TBS or TBST buffer after incubation in 3% hydrogen peroxide for 10 minutes. The non-specific reaction was blocked with 100–400 µl blocking solution for one hour at room temperature (5% normal goat or rabbit serum in TBS). The diluted primary antibody, Biotinylated-secondary antibody, and ABC reagent were sequentially added, and this was proceeded with incubation and rinsing with TBS and TBST after each reaction, in accordance with the manufacturer's instructions. The reaction results were visualized with NovaRed Kit (Vector Laboratories, Burlingame, CA). The sections were dehydrated and sealed in Permount (Fisher Scientific, Ottawa, Ontario) for observation. A single pathologist (EF) performed semi-quantative evaluation based on intensity of cytoplasmatic staining [0− no stain, 1+ weakly positivity (difficult to see) -3+ (prominent stain)] and then determined the percentage of positive cells (those with any degree of staining), as we described before [19]. Accordingly, the staining intensity index (SII) was calculated as the percent of positive cells x intensity/100.

### Determination of serum testosterone, gAOC, and 8-OHdG levels

An enzyme-linked immunosorbent assay (ELISA) kit specific to quantitative measurement of 8-hydroxy-2′-deoxyguanosine (Oxford Biomedical, Oxford, Michigan, USA) was used to measure the serum 8-OHdG levels according to the manufacturers' instructions. Each sample was tested in triplicate, with the standard run in duplicate. A portion of the manufacturer's provided standard was diluted to a concentration of 1.0 and 5.0 ng/mL in order to best approximate the total concentration of each sample, as previous test runs indicated that levels of 8-OHdG were low. Within-assay precision was tested and found to be greater than 96.6% while edge effects were tested and found to be negligible. In a similar manner, ELISA was undertaken to test serum testosterone levels according to the maker's instructions. (Alpha Diagnostic International, Cat. No. 1880, San Antonio, Texas, USA).

To measure serum gAOC, a test was carried out with BIOXYTECH® AOP-450™ Colorimetric Quantitative Assay Kit for Total Antioxidant Potential (Aqueous Samples) by *Oxis*Research™ (Catalog Number: 21053, Beverly Hills, California, USA), according to the manuals of maker.

### Data Analysis

A student t-test with a 5% significance level was used to measure differences is serum gAOC and serum testosterone between the A+ and A− or C groups. One-Way analysis of variance was used to identify variance in serum 8-OHdG levels between androgen groups. A Mann-Whitney test was used to test for significant differences in staining intensities between treatment groups. Univariate linear regression was used to measure if increases in serum testosterone concentration results in increases in OS in the surrogate tissues. This test was also used to measure if serum AOC correlated with the OS in the target tissues. Multivariate linear regression was used to analyze correlations in OS staining intensities between tissue sites within each individual rat. This form of analysis was also used to examine if there were inter-individual differences in OS staining between rats within each androgen group as well as to examine if there was a correlation between Prostatic OS and serum 8-OHdG or serum AOC. Heterogeneity was tested using both a Breusch-Pegan test and a White test. Data was analyzed by STATA software (Statacorp LP, College Station, Texas, USA).

## Results

The eighteen adult male rats were divided into three treatment groups. The first group underwent a bilateral trans-scrotal orchiectomy and was labeled “A−” due to their lack of androgen. The second group was given testosterone supplementation through a subcutaneous pellet and was labeled “A+” which indicated their excess androgens. The last group, “C” had no change in androgen levels and was thus the control group. Systemic and tissue (prostate, salivary glands and hair follicles) OS levels in each rat were measured using 8-OHdG staining and measuring. The 8-OHdG biomarker was used because the half-life of reactive oxygen species is extremely short (milliseconds) and therefore common measurements of OS status are based on the detection of their more sustained effects on other molecules. Since this study was conducted with the aim to study the concept of individualized approaches to prostate cancer prevention we elected to study a marker for OS induced mutagenesis. Among ROS-induced forms of DNA damage, 8-OHdG is typical and most commonly used as a marker for quantitative analysis [20]. As well, systemic AOC and testosterone were measured and examined.

### Androgen effect on OS

Serum testosterone levels of the three treatment groups were significantly different (p<0.05) with the “A+” rats having the highest testosterone levels (mean = 8 mg/mL, median = 8.4 ng/mL, Tables 1 and 2) and the “A−”rats all having castrated levels (<0.5 ng/mL). This confirmed that we had indeed created groups with three different levels of androgens: castrated (A−), normal eugonadal (C) and increased (A+).

**Table 1:** Experimental data per individual rat.

Androgen Group		Rat 1	Rat 2	Rat 3	Rat 4	Rat 5	Rat 6
**A+**	Serum Testosterone (ng/mL)	8.5	9.2	8.4	5.8	9.69	6.3
	Serum 8-OHdG (ng/mL)	3.4	6.0	3.9	2.7	2.0	4.4
	Serum gAOC (Relative AOP450)	0.5	1.3	0.5	1.0	1.2	1.5
	Prostatic OS Staining Score	2	2	2	2	2	1
	DPC OS Staining Score	2.5	1	2	1.5	2	2.5
	Salivary Gland OS Staining Score	3	2	2	3	−	1
**A−**	Serum Testosterone (ng/mL)	0.2	0.2	0.2	0.2	0.2	0.2
	Serum 8-OHdG (ng/mL)	3	4.6	4	2.8	4.6	3.6
	Serum gAOC (Relative AOP450)	1.3	0.4	0.4	0.7	1.1	0.9
	Prostatic OS Staining Score	0	0.5	1	0	1	1.5
	DPC OS Staining Score	0.5	0.5	1	0.5	1	1
	Salivary Gland OS Staining Score	1	2	1	1	1	1
**C**	Serum Testosterone (ng/mL)	0.8	0.8	0.8	0.7	0.8	0.8
	Serum 8-OHdG (ng/mL)	3.5	4.3	2.5	3.5	2.8	1.4
	Serum gAOC (Relative AOP450)	1.2	0.6	0.7	0.4	0.5	0.8
	Prostatic OS Staining Score	1	2	1.5	1.5	1.5	0.5
	DPC OS Staining Score	1	2	2	2	1	2
	Salivary Gland OS Staining Score	1	1	1.5	1	2	1.5

Please note the lack of correlation between serum testosterone level and serum 8-OHdG levels and tissue expression of OS.

**Table 2:** Mean, median and range of several factors measured.

Group	Mean, median and range of serum testosterone (ng/ml)	Mean, median and range of serum 8-OHdG (ng/ml)	Mean, median and range of serum gAOC (relative AOP450)
**A+**	8.0, 8.5, 5.8–9.69	3.7, 3.6, 2.0–6.0	1.0, 1.1, 0.45–1.46
**A−**	0.2, 0.2, 0.17–0.24	3.8, 3.8, 2.8–4.6	0.8, 0.8, 0.39–1.32
**C**	0.8, 0.8, 0.74–0.81	3.0, 3.2, 1.4–4.3	0.9, 0.7, 0.37–1.23

Serum testosterone, 8-OHdG and global anti-oxidative capacity (gAOC) per experimental group. Values were rounded to the nearest tenth for all measurements and only the ranges are shown with 2 decimal places to show the variation in values.

**Figure 1:**
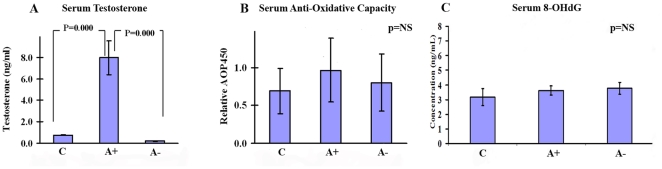
Serum testosterone (A), anti-oxidative capacity (B), and 8-OHdG levels (c) per treatment group. Results are shown as mean +/− S.D. P values were calculated using a two-tailed t-test.

**Figure 2:**
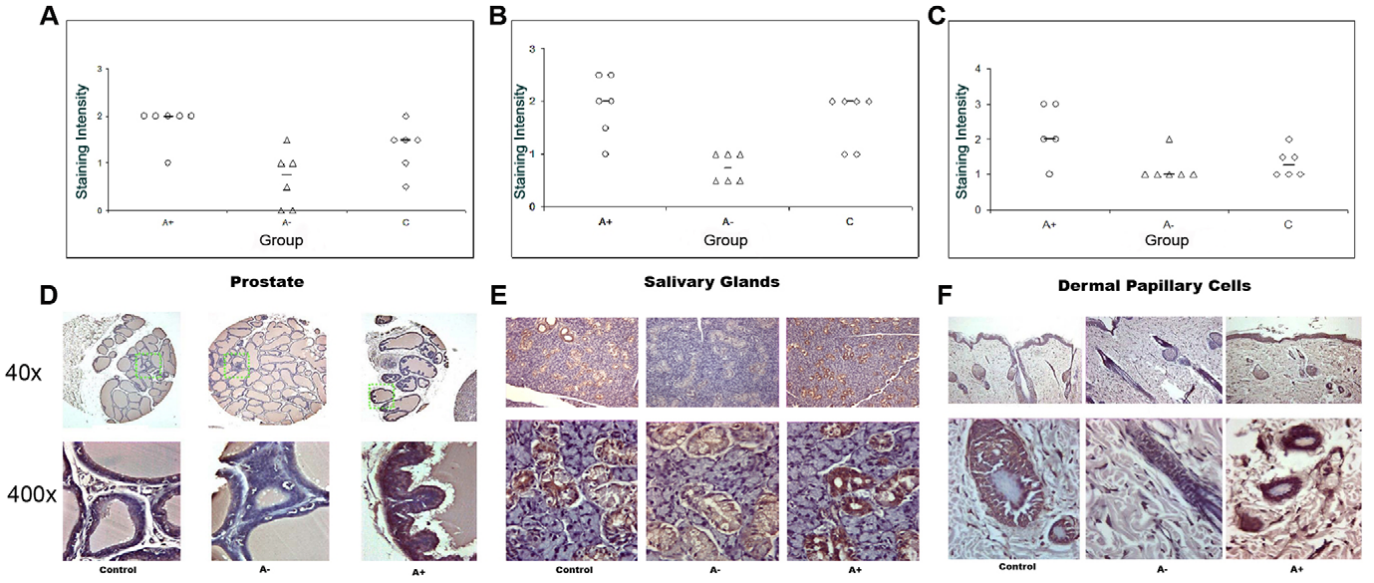
Oxidative stress in androgen regulated target tissues: Prostate (A&D), salivary glands (B&E) and hair follicles-DPC (C&F). A–C: Graphs showing the individual staining intensity according to treatment groups (A+: circles, A−: triangles, C: diamonds). Median values are shown as a line. D–F: Representative 8-OHdG immunostaining under low (×40) and high (×400) magnification.

Despite these significant differences in serum testosterone levels, the serum 8-OHdG levels were not significantly different between the groups (p>0.05, ANOVA test) indicating that androgens do not affect *systemic* OS (Figure 1). In contrast, OS levels in the prostate, salivary glands and DPC showed a positive relationship with serum testosterone concentration (p>0.05, Mann-Whitney Test; Figure 2). In all three target tissues, there were statistically significant differences between the “A+” and “A−” rat groups with regards to 8-OHdG staining, with the androgen supplemented rats staining more intensely (p = 0.019). The median staining intensity for the hair follicles of androgen-deprived rats was 1, a much lower grade than the median for the androgen supplemented rats which was 2. Similar results were found for the median staining intensities for the hair follicles and prostate. In both tissues, the “A+” rats had the highest median staining grades and the “A−” had the lowest. (Tables 1 and 2 & Figure 2) Therefore, it can be deduced that the OS in the three target tissues increases as the level of androgens in the body increase.

**Figure 3:**
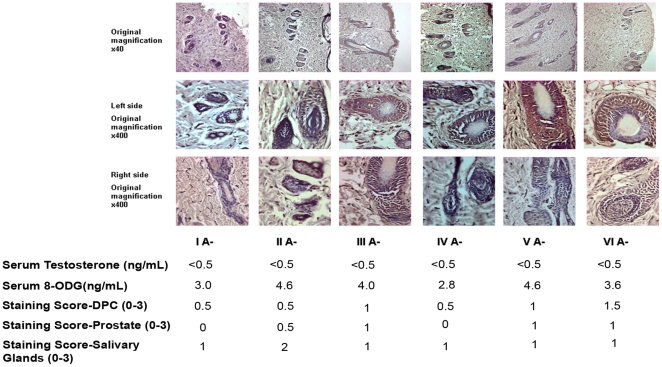
Inter-individual differences and intra-individual similarities in the castrated rats. Inter-individual variability in serum OS and target tissue 8-OHdG staining in rats with similar (castrated) levels of testosterone.

Surprisingly, we discovered that there were inter-individual differences in 8-OHdG staining intensities between rats with similar serum testosterone levels (p<0.05). Even in the castrated rats, which all had almost no serum testosterone (<0.5 ng/mL), we found that heterogeneity existed between their OS staining grades in the prostate and surrogate tissues. (Figure 3) These differences in OS could not be predicted by Serum 8-OHdG (p<0.05) (Figure 3), thus suggesting that prostatic OS varies between individuals with similar androgen levels.

### Target Tissue Relationships

After controlling for androgen group and testing a linear combination of parameters, we found that the DPC were very effective in predicting the OS of the prostate (p = 0.009). Furthermore, the salivary glands were also found to be significantly effective in measuring the OS of the prostate (p = 0.001). Our findings purport that the OS of the prostate is very similar to the OS of the hair follicles and salivary glands in each individual, and that these tissues may be predictive as surrogates for the OS status of the prostate.

### Relationships between serum anti-oxidative capacity (AOC) and prostatic OS

Finally, we examined if systemic AOC correlates with OS status in androgen regulated tissues in general and the prostate in particular. Unlike systemic OS (as reflected by 8-OHdG levels) there was a negative correlation between serum AOC levels and OS stress staining levels in the 3 target tissues (p<0.01). This implies that systemic manipulation of OS is not only efficient in reducing prostatic OS, but also OS of the DPC and salivary glands. The DPC and salivary glands can therefore be used as surrogate instruments to measure the efficiency of anti-oxidative PC prevention strategies on an individual basis.

## Discussion

Our results indicate that each individual has a unique pattern of OS in his prostate that is mirrored in his hair follicles and salivary glands. An individual's prostatic OS cannot be predicted merely by serum testosterone levels, as even in the group of castrated rats inter-individual differences exist (Figure 3). Moreover, the oxidative capacity in the serum is equal between hosts with wide differences in their serum testosterone levels (Figure 1) suggesting that androgens do not affect systemic OS. Therefore, serum androgen levels and serum OS do not reflect prostatic OS status. In contrast, the OS in the hair follicles and the salivary glands is closely correlated with prostatic OS levels.

Hoque et al analyzed the serum levels of protein carbonyl groups (a marker of OS) in 1,808 PC cases and 1,805 controls nested in the prostate cancer prevention trail [21]. The study reported that despite the effect of reduced DHT levels by Finasteride, there were no significant associations between serum OS and PC risk. The findings of their study augment our hypothesis that, with respect to the promoting effects of androgens on OS in particular, the risk of PC development can not be predicted by blood measurements of OS related markers. However, as shown here, OS status in readily available androgen regulated surrogate tissues such as the hair follicles may accurately reflect that of the prostate.

The impact of androgens on salivary tissues is thus not limited to an influence on morphology [22]–[24], but also includes a broad spectrum of effects on glandular physiology [25], [26]. Androgen receptors have been identified in the salivary glands of males and females [27] with a higher concentration of nuclear androgen-binding sites in males [28]. It has been found that salivary glands actively up-take androgens from the plasma and rapidly metabolize them [29]. Indeed, androgens are known to be the primary mediators of sexual dimorphism in mouse salivary glands. Accordingly, Andrews and Bullock demonstrated that the enzymatic activity of alkaline phosphatase in male salivary glands is consistently stronger compared to that in female and in control androgen-insensitive *tfm/*Y mutant male mice [30]. Furthermore, several studies have shown that OS markers can be measured in the saliva [31], [32]. In addition to these morphological and physiological similarities between the salivary glands and prostate, our study found a close correlation between the OS status of these two tissues.

Androgens act on the hair follicle via the androgen receptor in dermal papillary cells (DPC). The DPC influence the other cells of the hair follicle by altering the production of regulatory substances such as growth factors and/or extracellular matrix components [33]. It has been shown that the graying hair follicle develops aging-relating OS and is target by “anti-aging” therapies [34]. A study by Minelli et al. recently discovered that the prostate also develops OS as natural part of the aging process [35]. Our study has found that the OS in these two tissues is similar and suggests that hair follicles may be able to be used as a surrogate tissue to the prostate when measuring OS.

Compared to normal subjects or patients with benign prostatic hyperplasia (BPH), patients with PC have a systemic imbalance in their OS/antioxidant status [36]. Increased levels of ROS within the prostate stimulate cell proliferation, induce somatic DNA mutations and promote genetic instability facilitating the development and progression of PC [2]. Apart from the pro-oxidative effect of environmental factors such as diet and inflammation, we [7], [37] and others [38] have shown that androgens increase the production of ROS in PC cells. Should the results of those studies prove robust, the current study has significant potential implications in the field of PC prevention.

OS may favor the induction of mutagenic processes within the prostate [39]. Attempts to modulate OS by anti-oxidative compounds are common practice in PC chemoprevention. However, the results of the SELECT study demonstrate no impact of Selenium or Vitamin E on the risk of PC, causing the termination of the study [13]. A potential explanation for this is the fact that the chemoprevention protocol, which was aimed to reduce OS, was not tailored to individuals. There was no assessment of the baseline prostatic oxidative status of the study participants before the administration of the compounds or placebo. Since different individuals have different profiles of OS, one would expect minimal or no effect of anti-OS compounds in subjects with lower baseline OS.

Alternatively, application of daily chemoprevention is expected to be more beneficial and to be accepted with higher compliance in subjects with high baseline levels of prostatic OS. However, there is no non-invasive method of determining prostatic OS. The ability to predict the prostatic OS in chemoprevention candidates without the need to biopsy the prostate is thus appealing. Recent research has provided strong evidence to suggest that the saliva can be used as an assay for several biomarkers. In fact, numerous studies have measured biomarkers for OS (8-OHdG) and total anti-oxidative capacity by just using the saliva [40], [41]. This would suggest that since our study has shown that the salivary glands can be used as surrogate tissue to the prostate, one may predict prostatic OS by simply testing the saliva without having to biopsy the gland itself. As well, as mentioned above, the OS of the DPC can be measured by using the hair follicle. Therefore, a simple plucking of the hair (as opposed to a skin biopsy) may also be used to predict prostatic OS. Due to the virtually painless nature of these diagnostic methods and the potentially large benefit of obtaining a prostatic OS profile of an individual, we emphasize the importance of the results of our research and the necessity for further study.

There are several limitations to the study. Sample size was small. However, this experiment was designed as a hypothesis-generating study only and serves as a solid basis for further study in humans. Another limitation of our study was that, due to the confines of our experimental model, the rats were not exposed to other factors that may result in OS of the prostate such as prostatic inflammation. Individuals exposed to these factors may have different OS levels in their surrogate tissues; however further study in humans, who unlike rats have intra-prostatic inflammation, is necessary to look at this. Lastly, we were unable to monitor rats over a long period of time and therefore, results reflect only an acute response to changes in androgen levels.

This hypothesis-generating *in vivo* study suggests that the examination of the OS of the DPC of the hair follicles may provide one with an individual signature of the OS status in the prostate. Clinical studies are needed to validate these findings in humans and their potential prognostic implication in the individualization of PC prevention and therapeutics.
